# Cell-Based Immuno-Biosensors Using Microfluidics

**DOI:** 10.3390/s26061970

**Published:** 2026-03-21

**Authors:** Briggs Pugner, Erik Petersson, Seedahmed Ahmed, Maha Mustafa, Justin Okoh, Yuhao Qiang

**Affiliations:** 1 Department of Engineering, University of Maryland Eastern Shore, Princess Anne, MD 21853, USA; bjpugner@umes.edu (B.P.);; 2 Department of Pharmaceutical Science, School of Pharmacy, University of Maryland Eastern Shore, Princess Anne, MD 21853, USA; jokoh@umes.edu

**Keywords:** immuno-biosensor, microfluidics, machine learning, point-of-care

## Abstract

Cell-based immuno-biosensors are novel platforms for studying immune responses of biological cells, with real-time insights more similar to physiological and pathological conditions. These systems utilize living immune cells as their main components, enabling them to detect disease-related biomarkers and cellular traits in a way that is often highly sensitive and label-free. Integration with microfluidics and organ-on-chip technologies has facilitated precise manipulational control over the cellular microenvironment. Not only has this resulted in high-throughput screening, but it also enabled smaller, more portable systems which can be used at the point of care. In this work, we review the recent advance in microfluidic cell-based immuno-biosensing associated with immune cells such as neutrophils, macrophages, T cell and dendrite cells. Some of the exciting developments include fusion with methods such as advanced imaging, electrical impedance sensing and application of machine learning to phenotyping. We will also elaborate on the issues related to the standardization of these systems, cell heterogeneity, and the challenges for translating these technologies for clinical application. Taken together, such integrated platforms have potential to fill the gap left in-between cellular immunology with biosensor engineering.

## 1. Introduction

Immuno-biosensors have been mostly used for immunological recognition elements, including antibodies, antigens, or immune cells, to detect specific target molecules by exploiting the high specificity of antigen–antibody or immune receptor–ligand interactions, with the resulting immune interactions being transduced into measurable physical or chemical signals [1]. Immuno-biosensors have emerged during the COVID pandemic due to their capability for rapid, sensitive, and point-of-care diagnostics [2]. Conventional immunology testing methods, such as ELISA, Western blot and PCR, are time-consuming and require expensive lab infrastructure and professional training [3]. Immuno-biosensors offer the possibility of real-time detection, making them highly promising tools for novel immunological testing.

Cell-based biosensors are analytical platforms that employ living cells as the recognition element and convert cellular responses to external stimuli into measurable signals. In contrast to the conventional molecular-based immune-sensors that primarily quantify specific binding events using biologically active substances such as enzymes, DNA, antigen, antibodies, cell-based biosensors can provide integrated functional and physiologically relevant information, such as pharmacology, cell physiology, toxicology within a intact cell [4]. Advances in sensing technologies, such as mechanical sensing, electrochemical sensing, piezoelectric and acoustic wave sensing, magnetic biosensing and optical biosensing, have greatly enhanced transduction and detection methods exploited in the development of cell-based immuno-biosensors. To clarify the complementary roles of these two approaches, Table 1 summarizes key differences in sensing elements, readouts, and biological context from molecular to organ/tissue levels.

This review aims to specifically present the existing cell-based microfluidic platforms that have been used for immuno-biosensing in the past years. The review provides an overview of different types of biosensors, exploring their unique applications and mechanistic approaches.

## 2. Principles of Cell-Based Microfluidic Immuno-Biosensors

**Figure 1:**
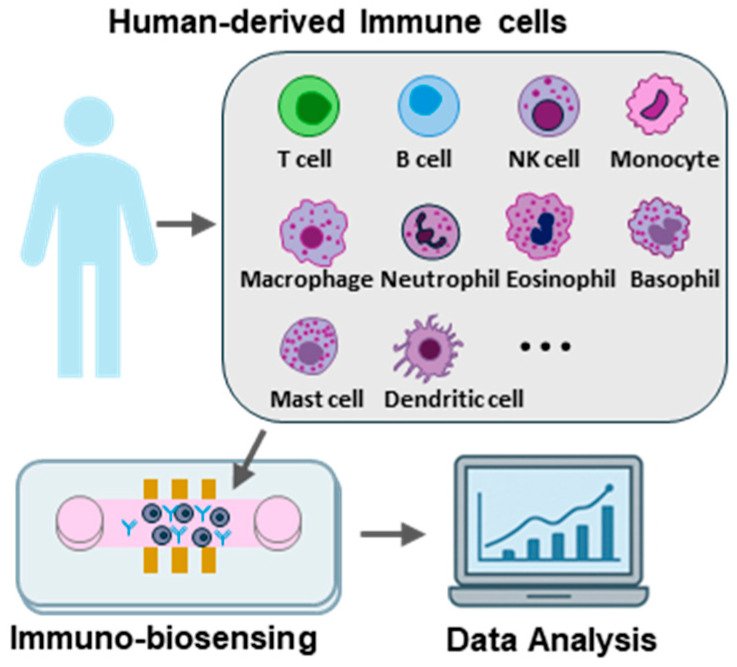
A schematic overview showing the workflow of microfluidic immuno-biosensors for human-derived immune cells.

Cell-based microfluidic immuno-biosensors exploit the natural immune recognition of intact living immune cells, which respond to specific stimuli, e.g., antigens, toxins, pathogens and drugs, external oxidative stress or mechanical stress, by altering their physiological, biochemical or biophysical properties. Those changes can be converted with measurable signals through a transducer, such as optical, electrochemical, or mechanical biosensors. The immune system comprises a variety of immune cellular populations, including T cells, B cells, macrophages, NK cells, dendritic cells, and monocytes, each distinguished by characteristic phenotypes and specialized functions. Therefore, researchers have employed distinct sensing modalities in the analysis of immune cells based on optical, electrical, mechanical, electrochemical readouts from cell behaviors, responses and interactions. Figure 1 shows a typical workflow: (1) Immune cells are first isolated from human samples (or generated/engineered, e.g., CAR-T/CAR-NK) and then introduced into a microfluidic or organ-on-chip platform that provides controlled delivery of stimuli and a defined microenvironment (flow, gradients, mechanical cues, and cell–cell contacts). Upon stimulation, immune functions such as activation, migration, cytokine secretion, degranulation, and cytotoxic interactions are elicited within the device. (2) These cellular responses are captured in real time using integrated sensing modalities (optical imaging/fluorescence, electrical/impedance, electrochemical, or mechanical/acoustofluidic readouts) and converted into quantitative signals. (3) Finally, the resulting datasets are analyzed, often with automated algorithms and AI/machine learning, to extract immune-state metrics and support downstream applications including immune monitoring, drug screening, and mechanistic studies.

## 3. Microfluidics as a Platform for Immuno-Biosensors

Microfluidics could provide a miniaturized and sensitive platform by integrating sample handling, immune-cell manipulation, and signal transduction within precisely controlled microscale environments. It can be innately incorporated with advanced detection technologies such as acoustics, optics, and electric fields to leverage physical characterization. Therefore, microfluidic platforms have many advantages over the traditional immuno-assays, e.g., low consumption of reagents, improved automation and portability, and real-time monitoring. Microfluidic platforms can be fabricated using various biocompatible materials, such as polydimethylsiloxane (PDMS), thermoplastics (e.g., polymethyl methacrylate (PMMA) and polycarbonate), glass and hydrogels, which could provide analytical performance for biosensing applications in living systems. In addition, the advancement in microfabrication technology could significantly enhance the design of microfluidic chips with desirable microfeatures for biosensing applications [5]. Most importantly, microfluidic architectures enable the spatiotemporal control of the cellular microenvironment (e.g., chemical gradients, localized mechanical or electrical stimulation, and compartmentalization) that is difficult to achieve in bulk measurements, making them particularly well-suited for single-cell immunology and functional immune profiling. Current microfluidic immuno-biosensors can be categorized based on their operational and immunological readout formats (see Table 2).

### 3.1. Continuous-Flow Microfluidic Immunoassay Chips

Continuous-flow devices use microfluidic channels and chambers to achieve automatic immunoreactions (e.g., immunocapture, washing, and detection) and measurement of cellular secretion under controlled flow [6]. These platforms are widely used for rapid quantification of soluble immune biomarkers (e.g., cytokines, chemokines, antibodies), pathogen antigens, and inflammatory markers of immune cells. Their key advantages include lower reagent use, faster binding kinetics due to short diffusion paths, and streamlined workflows that can reduce hands-on steps and enable point-of-care operation. Moreover, continuous-flow chips can be coupled to optical readouts (fluorescence, absorbance), electrochemical detection (amperometry/voltammetry), or impedance-based sensing, supporting multiplexed panels when combined with spatially patterned capture regions [11]. This method is limited in their reproducibility due to the potential impact of complex sample matrices and environmental conditions. Moreover, it can be challenging due to contamination risks introduced by external fluidic interfaces.

### 3.2. Droplet Microfluidics for Compartmentalized Single-Cell Immunoassays

Droplet microfluidics partitions samples into picoliter-to-nanoliter droplets that function as isolated microreactors, often enabling one-cell-per-droplet formats [7]. For immuno-biosensing, droplet platforms are particularly valuable for single-cell functional assays such as cytokine secretion profiling, immune-cell activation screening, antibody discovery, and assessment of heterogeneous responses within mixed populations. By physically isolating single cells, droplet systems minimize crosstalk, increase effective analyte concentration, and allow high-throughput screening with tight control over exposure conditions. Typical readouts include fluorescence-based assays using labeled reporters or bead-based capture, with downstream decoding via imaging or sequencing-compatible barcoding strategies. However, this microfluidic technique is limited to its device complexity, and emulsion droplet stability, and interfacing to downstream analysis.

### 3.3. Microwell and Digital Immunoassay Platforms

Microwell arrays, microchambers, and microarray-style compartmentalization are frequently used to capture single cells or single molecules and perform parallel immunoassays in a stationary condition [8]. In immuno-biosensors, these platforms enable high-throughput single-cell phenotyping and secretion measurements, which can support “digital” immunoassay principles (counting discrete events) to improve analytical sensitivity. Compared with bulk immunoassays, microwell and digital formats can achieve improved limits of detection through compartmentalization, reduce assay volumes, and facilitate multiplexing by spatial encoding. Optical imaging (fluorescence/chemiluminescence) is common for the readout measurement, although electrochemical and impedance-based approaches can also be integrated depending on the device design. The limitation of this microfluidic assay is that it is required to incorporate a high capacity of imaging system and the workflow complexity. Due to the limited medium volume, the platform is also not good for long-term cell culture biosensing.

### 3.4. Organ-on-Chip and Immune-Organoid-Based Sensors

Organ-on-a-chip systems mimic the microarchitecture, fluidic dynamics, and tissue–tissue interfaces of human organs, enabling precise control of immune cell–tissue interactions under dynamic conditions [9]. In parallel, immune organoids recapitulate three-dimensional immune microenvironments, such as lymphoid structures or tumor–immune niches, allowing the study of complex cell signaling and immune responses in vitro. These systems are useful for functional studies such as leukocyte adhesion and transmigration, inflammation and barrier disruption, immune–tumor interactions, immunotoxicology, and pharmacological modulation under flow, which makes them strong candidates for integration with immuno-biosensing modules [12]. Their main advantage is improved physiological relevance compared with static culture and traditional assays, enabling measurement of integrated immune behaviors over time. When coupled with biosensing modalities, including electrical impedance, optical, or electrochemical sensors, these systems can provide quantitative and time-resolved measurements of immune activation, cytokine secretion, and cytotoxicity. Together, organ-on-a-chip and immune-organoid-based immune-biosensors offer a powerful framework for personalized immunological assessment, drug screening, and mechanistic investigation of immune pathophysiology. The main limitations are the higher cost, complexity in standardization, and long setup time compared to the conventional flask/Petri dish assays.

### 3.5. Microfluidic Sample Preparation and Sorting Modules as Enabling Components

Many microfluidic immuno-biosensors incorporate upstream modules for immune-cell enrichment, washing, and sorting (e.g., inertial microfluidics, dielectrophoresis, acoustofluidics, magnetophoresis) [10]. These modules improve sensitivity and robustness by concentrating rare targets (e.g., specific immune subsets, circulating biomarkers, pathogens, extracellular vesicles) and reducing matrix interference before detection. When integrated on-chip, these systems enable automation and scalable operation, supporting continuous “sample-in/answer-out” immuno-biosensing workflows. Nevertheless, these methods remain constrained by device-specific tuning requirements and limited reproducibility, driven by sample-to-sample variability and the potential for cell stress during on-chip manipulation.

## 4. Recent Advances in the Application of Cell-Based Microfluidic Immuno-Biosensors

Microfluidic cell-based immuno-biosensors integrate living immune (or engineered) cells with miniaturized fluidic control and on-chip signal transduction to quantify functional immune responses in a rapid, low-volume format. By regulating flow, shear stress, chemical gradients, and cell–cell interactions, microfluidics can recreate key aspects of physiological microenvironments while enabling automated sample handling and multiplexed detection. In this section, we highlight recent application-driven progress in these platforms, focusing on how microfluidic integration has expanded their utilities in pathogen detection, cancer diagnostics, drug discovery and screening, and personalized immune profiling.

### 4.1. Pathogen Detection

**Figure 2:**
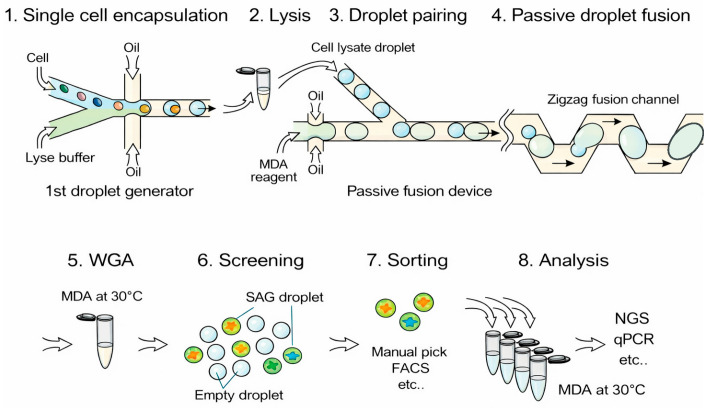
Single-cell genome analysis process using droplet microfluidics. Reproduced from ref. [17] under the terms of the Creative Commons Attribution 4.0 International License (CC BY 4.0).

The influence of pathogens on cellular immune responses is more complicated than that at the antibody level. For instance, the activation of lymphocytes leads to multiple cellular transformations which include cell growth and morphological changes and genetic profile modifications and protein production [13]. Therefore, the natural pathogen recognition abilities of living immune cells make cell-based immune biosensors suitable for fast and sensitive pathogen detection. These biosensors depend on immune cells, such as macrophages and engineered cell lines to detect pathogens through their antigen and pathogen-associated molecular pattern recognition receptors. The cells produce detectable signals through cytokine secretion and metabolic changes and bioelectrical responses which get converted into measurable outputs through optical and electrochemical and electronic systems [14,15]. This approach provides unique benefits because it enables real-time detection of complete pathogens while evaluating their biological functions which makes it suitable for medical testing and food safety checks and environmental monitoring. The combination of droplet microfluidics with fluorescence and nucleic acid amplification and antibody-based assays enables pathogen detection through the creation of picoliter-sized droplets that function as independent microreactors for single-cell analysis (Figure 2) [16,17].

### 4.2. Cancer Diagnostics

**Figure 3:**
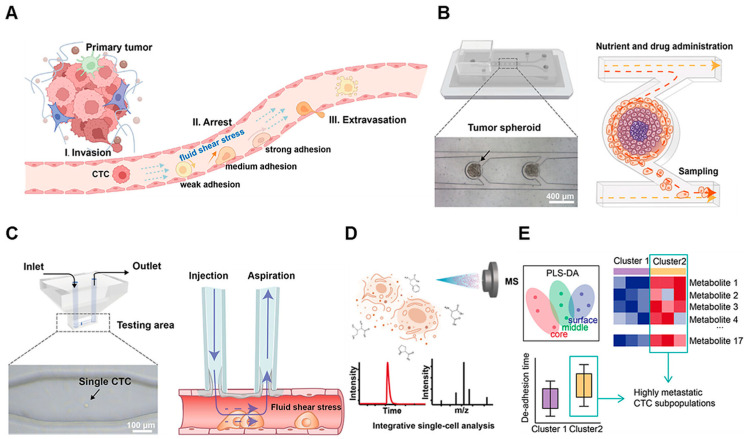
Integrated CTC behavior and metabolic analysis. (**A**) A schematic diagram showing the CTCs’ arrest behavior in vasculature. (**B**) The section of the microfluidic chip cultured with tumor spheroids. (**C**) The section of the microfluidic chip mimicking the CTC arrest behavior in the transient vascular system. (**D**) MS metabolic analysis. (**E**) Integrative single-cell analysis. Reproduced from ref. [22] under the terms of the Creative Commons Attribution 4.0 International License (CC BY 4.0).

Microfluidic platforms have recently been integrated with the immuno-biosensors for the cancer diagnostics, offering improved sensitivity, faster analysis, reduced sample volumes, and compact device design [18,19]. In most reported applications, microfluidics are primarily employed for automated sample preparation, such as blood cell separation [20]. Beyond this, many systems utilize microfluidic channels to facilitate the detection of specific antibodies or proteins, thereby improving the efficiency and reliability of biomarker assays [21]. For example, Hou et al. developed a microfluidic system that links metastatic behavior with metabolic heterogeneity of single circulating tumor cells, identifying high-metastatic subpopulations and showing that 5-fluorouracil reduces their arrest ability via metabolic reprogramming (Figure 3) [22]. Chikkaveeraiah et al. developed an amperometric electrode microfluidic array by combining a PDMS-based chip with a screen-printed carbon array modified with glutathione-coated gold nanoparticles functionalized with specific antibodies, enabling the detection of prostate-specific antigen and interleukin-6 (IL-6), biomarkers of prostate cancer, through immunoaffinity binding on the electrode surface [23]. McGrath et al. developed a label-free electrical impedance cytometry to quantify phenotypic heterogeneity for rapidly stratifying tumorigenicity [24].

### 4.3. Drug Discovery and Screening

Cell-based immune-biosensors integrated with microfluidics are emerging as high- throughput and low-volume platforms for evaluating drug efficacy and safety [25]. By combining primary or engineered immune cells with dynamic on-chip regulation of fluids, gradients, and co-cultures, these systems provide rapid, multiplex readouts that more closely resemble human biology as compared to conventional static assays [26]. Recently, organ-on-a-chip (OOC) systems have been established as robust in vitro platforms for the analysis of immune cells and drug screening [27,28,29,30]. By replicating human microenvironments (vascular flow, tissue–tissue interfaces, matrix mechanics, and cytokine gradients), they allow tunable investigations of leukocyte trafficking, antigen presentation, tumor–immune crosstalk and engineered cell therapies (e.g., CAR-T) under physiologically relevant shear flow. Combined sensors and live-imaging provide real-time readouts for activation, cytotoxicity, cytokine release and toxicity; patient-derived systems enhance clinical relevance for efficacy and safety testing. OOC models predict human outcomes with higher accuracy than many organ-specific animal models for efficacy and safety (e.g., hepatic or cardiac liabilities) testing and can significantly contribute to the reduction in animal numbers but currently serve as complementary systems rather than outright replacements of animals for questions which require integrated, long-term whole-organism physiology.

### 4.4. Personalized Medicine

**Figure 4:**
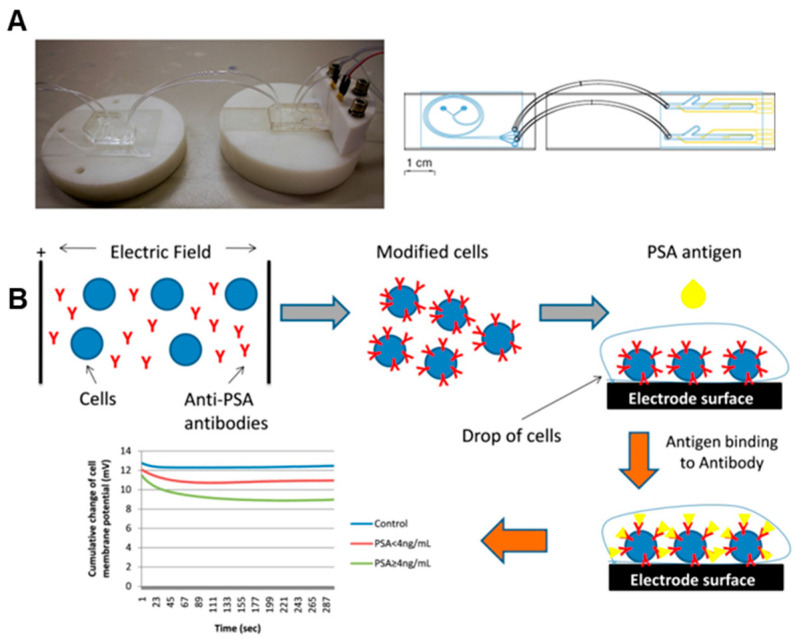
(**A**) An integrated microfluidic biosensor for assessing CD4^+^ T-cells. (**B**) Schematic diagram showing the workflow of an ultra-rapid point-of-care (POC) biosensor for prostate-specific antigen detection in human serum. (**A**,**B**) are reproduced from references [31,32], respectively, under the terms of the Creative Commons Attribution 4.0 International License (CC BY 4.0).

Cell-based immune biosensors constitute a versatile analytical platform for evaluating functional responses of patient-derived immune cells or molecules, which makes it a promising approach for personalized medicine due to their ability to directly utilize immune cells as functional sensing elements. These biosensors harness the natural responsiveness of immune cells, such as T cells, macrophages, or B cells, to detect and quantify biological signals that reflect an individual’s immune status or disease state. Because most immune cells can be immobilized and are readily accessible in peripheral blood, they offer a convenient and minimally invasive source for sensor development. For instance, Bialas et al. developed an electrochemical impedance spectroscopy sensor within a microfluidic device, which enables electrode functionalization and specific detection of patient-derived CD4^+^ T cells (Figure 4A) [31]. Moreover, engineered cells can also be employed to detect specific molecules secreted or generated by immune cells, enabling more targeted and sensitive detection of immune activity. For example, Mavrikou et al. developed a point-of-care biosensor for the detection of prostate-specific antigen (PSA) in human serum using a cell-based bioelectric recognition approach (Figure 4B) [32]. The researchers engineered Vero cells by electro-inserting anti-PSA antibodies into their membranes, enabling the direct transduction of antigen–antibody interactions into measurable changes in membrane potential. The resulting biosensor achieved label-free, real-time PSA quantification within five minutes, demonstrating high sensitivity, particularly in the clinically significant low-PSA range [32].

## 5. Recent Advances in Engineering Approaches and Innovations

Recent progress in cell-based immuno-biosensors has been driven by coordinated advances across multiple engineering approaches, including the biomimetic culture platforms, signal transduction and computational interpretation. In this section, we synthesize key innovations that collectively enable next-generation immune biosensing, including physiologically relevant model systems (organ-on-a-chip and immune organoids), diverse sensing modalities (optical, electrical/impedance, and acoustofluidic-enabled approaches) that convert immune function into quantitative signals, the incorporation of engineered immune cells as programmable sensing elements, and AI/machine learning methods for extracting robust phenotypes from complex, high-dimensional data. We further highlight the growing integration of single-cell and multi-omics workflows within microfluidic biosensors as an emerging direction for resolving immune heterogeneity and connecting functional readouts to underlying molecular mechanisms.

### 5.1. Incorporating Engineered Immune Cells in Cell-Based Immuno-Biosensors

**Figure 5:**
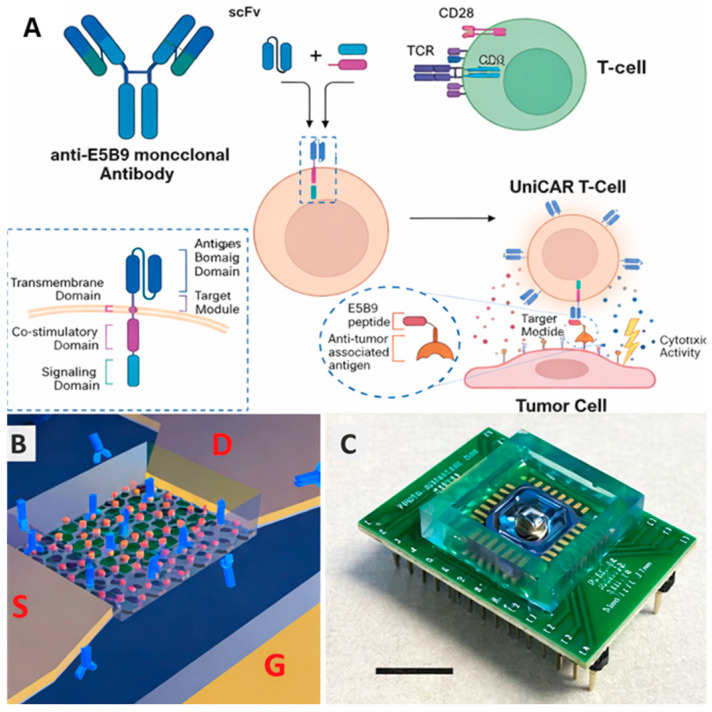
(**A**) CAR architecture and the working concepts of the UniCAR system. (**B**) A schematic of the biosensing platform formed from silicon honeycomb nanowires (SiNWs). Labels: S = Source, D = Drain, G = Gate. (**C**) Photographic image of the SiNW biosensor in a package. Scale bars, 10 µm. Reproduced from ref. [36], under the terms of the Creative Commons Attribution 4.0 International License (CC BY 4.0).

Organ-on-a-chip and immune-organoid systems offer significant promise as the next-generation platforms for immune biosensing by integrating physiological relevance with real-time functional readouts. A major recent advance in this field is the incorporation of living and engineered immune cells as the sensing interface, transforming conventional immunoassays into “active” biosensors that report functional immune behaviors (e.g., activation, degranulation, cytotoxicity) rather than only target binding. Immune cells such as macrophage, neutrophil, monocyte and dendritic cell can be used in biosensors due to the innate ability of these cells to target pathogens, toxins or antigens. These immune cells could also be used as an alternative or adjuvant to the synthetic receptors (e.g., antibodies, aptamers) employed in classical immunoassays in order to develop a living sensing interface with powerful and autonomous design for complex biological cues. Pizziconi et al. developed a hybrid, cell-based immune-biosensor that integrated living mast cells with microfabricated thermoelectric transducers to enable real-time detection of IgE-mediated antigens [33]. The mast cells were functionalized with preselected monoclonal IgE antibodies on their surface, allowing specific antigen recognition. Upon antigen binding, IgE–antigen interactions induced mast cell activation and degranulation, processes that generate measurable exothermic heat detectable by the thermoelectric transducer [33]. Mizumoto et al. designed a dendritic cell–based biosensor system to identify new immunostimulatory compounds through high-throughput functional screening using an IL-1β promoter–driven fluorescent reporter [34]. On the other hand, with the rapid development of engineered immune cells such as CAR-T and CAR-NK cells, this type of immuno-biosensor could also be adapted for evaluating the binding efficiency and functional characteristics of these engineered cells. For example, Lee et al. innovated a immunological synapse biosensor based on the FRET for the assessment of CAR-T cell functionality [35]. Nguyen-Le et al. developed a label-free silicon-nanowire field-effect transistor (SiNW FET) biosensor that is capable of detecting and quantifying engineered CAR-T cells from wild-type T cells at single cell resolution (Figure 5) [36]. Kim et al. developed a B cell–based biosensing and sorting platform that enables precise immobilization of B cells into well-defined single-cell arrays over large areas through surface biotinylation and specific protein adsorption based on the fluorescence measurement [37]. Similarly, Shanehbandi et al. developed a surface plasmon resonance-based immune-biosensor for the label-free detection of CD20^+^ B cells using an anti-CD20 monoclonal antibody immobilized on gold electrodes [38].

### 5.2. Sensing Modalities for Cell-Based Immuno-Biosensors

#### 5.2.1. Optical Readouts in Microfluidic Immuno-Biosensors

Optical approaches have been extensively used for cell-based immuno-biosensors, including fluorescence microscopy [39], fluorescence resonance energy transfer (FRET) based synapse sensors [35,40], and phase microscopy or Raman microscopy [41]. Beyond these direct imaging methods, microfluidics provides a powerful platform for the preparation of optical transducers to precisely engineer plasmonic, fluorescent, and structural color probes that could be favorably embedded into immuno-biosensors. Wang et al. reviewed how microfluidic channels enable precise control of metal nanoparticle size and shape for the generation of tunable plasmonic surface plasmon resonance/surface-enhanced Raman scattering substrates of optical biosensors [42]. Moreover, at the system level, smartphone-based imaging paired with machine learning has emerged as a compelling technique for reading out microfluidic optical signals at the point of care, with convolutional neuron networks compensating for limited signal-to-noise, small field of view, and user to user variability on low-cost hardware. Methods such as mHealth platforms, which already analyze fluorescence, colorimetric, and lens-free images of microfluidic assays, could be adapted to cell-based immuno-biosensors for decentralized monitoring of immune status, though challenges around optical material stability, regulatory-grade validation, and standardized phone-compatible hardware remain [43].

#### 5.2.2. Electrical and Impedance Readouts

Electrical measurement, particularly electrochemical impedance spectroscopy (EIS), can provide a unique label-free means to monitor both biorecognition and functional cell responses in microfluidic immuno-biosensors. Changes in interfacial charge transfer resistance and double layer capacitance can encode the complexes of antigen–antibody or cell–cell interactions, and link these impedance parameters to electrode geometry, nanomaterial coatings, and antifouling polymers [44]. For most of the cell-based immuno-biosensors, large receptors and whole cells are loaded within a limited Debye length on the order of a few nanometers, making surface engineering and the choice of Faradaic versus non-Faradaic operation critical to sensitivity and specificity. At the device level, microfluidic cell-based electrical biosensors typically culture mammalian cells onto microelectrode arrays or field effect structures, allowing for the continuous monitoring of changes in cellular morphology, adhesion, and viability under defined flow conditions [45]. Kiilerich Pedersen and Rozlosnik demonstrated that such systems support applications ranging from virus-induced cytopathic effect assays to chemotaxis and transmigration studies, effectively translating complex cell behavior into electrical impedance readouts [46]. Disease focused implementations, such as a Zika virus IDE immune-biosensor with a linear response from 10 pM to 1 nM and a reported limit of detection of 10 pM [47], and a spiral PCB EIS platform operating over 100 kHz–3 MHz for hepatocellular carcinoma and cirrhosis [48], illustrate how frequency-dependent impedance can distinguish specific antigen binding or pathological tissue states. However, these systems also have recurring bottlenecks, such as fouling, long-term stability, signaling drift, and the lack of standardized metrics for clinical validation.

#### 5.2.3. Acoustofluidic Actuation and Sensing

Acoustofluidics, particularly devices based on surface acoustic waves (SAWs), provides a contactless and controllable technique for manipulating cells, emulsion droplets, and molecular carriers within microfluidic channels, which is highly feasible for preserving viability in immune cell assays. Acoustic radiation forces and streaming flows can impose well-controlled shear stress, focus particles, or sort droplets without moving mechanical parts, allowing for precise control over the microenvironment experienced by immune cells on chip. Kim et al. demonstrated that Rayleigh-type traveling SAWs in a PDMS microchamber can generate defined shear stress on suspended NK92 cells at approximately 46.7 dyne/cm^2^ while maintaining a biocompatible temperature rise, with SAW derived shear elevating intracellular calcium and, especially when combined with PKC activation, enhancing cytotoxic function [49]. Acoustic forces can also improve the immuno-assays by overcoming diffusion limitations. In a focused SAW platform using FIDTs, Li et al. drive leaky SAWs to rapidly concentrate functionalized microbeads into a capture zone, achieving IgG sandwich immunoassays in approximately 20 s with detection limits down to about 100 pg/mL by converting acoustic focusing into enhanced binding and signal generation [50]. At the single-cell level, Zhong et al.’s CIAMAP system utilized standing SAWs to deflect and pair droplets containing NK92 and K562 cells, boosting productive effector–target pairing from Poisson limited fractions to roughly 80% and enabling high-throughput imaging, flow cytometry, and RNA seq of cytotoxic interactions. Taken together, acoustics serves as an integrated on-chip actuator that defines the mechanical niche of immune cells, concentrates rare targets, and solves droplet pairing bottlenecks, positioning acoustofluidic modules as powerful companions to optical and electrical readouts in next-generation cell-based microfluidic immuno-biosensors [51].

### 5.3. Integration with AI/Machine Learning for Data Interpretation

**Figure 6:**
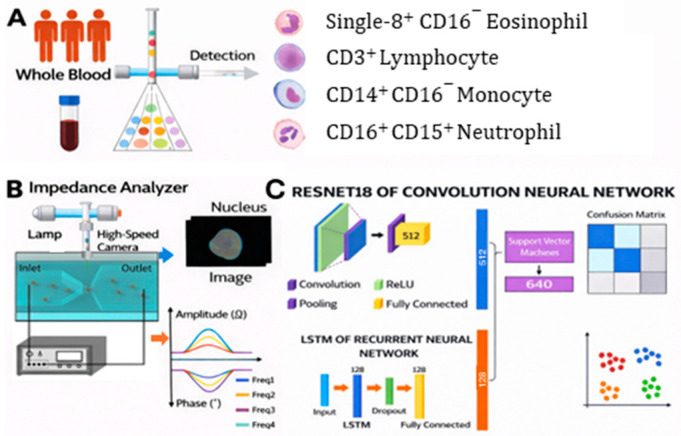
Workflow for leukocyte differential using imaging-impedance cytometry and deep learning. (**A**) Cell sorting using fluorescence-activated cell sorting method. (**B**) Schematic diagram showing the simultaneous fluorescent imaging and electric impedance measurement using the microfluidic device. (**C**) Data analysis using CNN (fluorescence) and RNN (impedance) models combined with an SVM for four-part leukocyte differentiation. Reproduced from ref. [56], under the terms of the Creative Commons Attribution 4.0 International License (CC BY 4.0).

The integration of Artificial Intelligence (AI) or machine learning technologies with immuno-biosensors could greatly improve the efficiency of data analysis, identification of subtle patterns and interpretation of high-dimensional or multiplexed data, especially for large and noisy datasets [52]. Through combination with cellular imaging methods, the transient dynamic process of immune cells could also be detected using deep learning-assisted object tracking and image classification methods [53]. Wang et al. developed a label-free impedance flow cytometer that employs a recurrent neural network (RNN) to classify electrical impedance profiles of single cells, enabling fast and automated differentiation of leukocyte subtypes [54]. Huang et al. successfully utilized a pre-trained deep convolutional neural network model (ResNet-50) to classify activated vs. inactivated neutrophil cells [55]. Similarly, Chen et al. developed a combined imaging and impedance flow cytometer for rapid immune-state monitoring by classifying white blood cells and neutrophil activation states using ResNet-50 (Figure 6) [56]. Pavillon et al. developed a label-free optical microscopy approach combining quantitative phase imaging and Raman spectroscopy to monitor activation of individual macrophages without the use of stains or contrast reagents [57]. They extracted morphological and molecular features of single cells under different stimulation/inhibition conditions, then applied machine learning classification to distinguish activation states with high sensitivity [57]. Hourani et al. developed a computational framework for macrophage phenotype identification that relies solely on intrinsic autofluorescence signals rather than any antibody-based staining [58]. Carrasco Yagüe et al. introduced an innovative framework for noninvasive, real-time monitoring of cellular dynamics by integrating EIS with machine learning-based analysis [59]. Using microelectrode arrays, the authors continuously recorded EIS signatures from live cell cultures and trained regression and classification models to extract key parameters such as cell density, diameter, and substrate coverage [59]. A nano biosensor platform has been designated to monitor tumor-derived immune cell responses associated with ovarian cancer progression, which integrates functionalized nanomaterials with electrochemical and optical transduction techniques to detect immune-related biomarkers released during early tumor development [60].

### 5.4. Multi-Omics and Single-Cell Analysis Within Microfluidic Biosensors

Due to the capacity to address cellular heterogeneity, single-cell assays based on microfluidic biosensors yield more accurate multi-omics analysis [61]. Classic droplet microfluidics has been intensively used for isolation of single cells and co-encapsulates them with barcoded beads in nanoliter droplets, creating independent compartments that minimize cross-contamination for the RNA sequencing and spatial multi-omics [62]. This technology is broadly deployed in commercial platforms, such as the 10× Genomics Chromium system [63]. Some other microfluidic methods are also used for the isolation of single cells, such as valve-based isolation method [64,65], microwell-based isolation method [66], and dielectrophoresis-trapping method [67], etc. Microfluidic single-cell sequencing assays have greatly advanced our understanding of the functions and molecular characteristics of immune cells. For instance, Zimny et al. utilized a hydrogel droplet microfluidic system to encapsulate single lymphoblast cells in alginate microparticles, extract individual nucleic acid strands, and conduct further genomic DNA analyses [68]. Lareau et al. facilitated a high throughput droplet-microfluidic platform for measurement of transposase-accessible chromatin at single-cell level [69]. Elizabeth et al. developed a microengraving system for the concurrent multiplexed interrogating multiple phenotypes of individual human peripheral blood mononuclear cells based on their secreted cytokines, antigen-specific antibodies and surface-expressed biomarkers [70]. A more comprehensive review of how single-cell immunology benefits from microfluidic biosensors and related technologies can be found in the review by Jammes et al. [71].

## 6. Challenges and Limitations

The cell-based microfluidic immune-biosensors, which are predominantly established on the specific antibody recognition elements, exhibit not only sensitive and miniaturized detection of protein moieties (e.g., pathogen and cytokine) in complicated biological specimen but also show good potentials in various other fields such as tissue engineering for immune organ mimicking purpose. However, challenges remain in maintaining biocompatibility and long-term stability of immune cells, preventing contamination and integrating living cells into portable, robust microfluidic devices. Incorporating physiological flow conditions is critical for cell-based microfluidic immuno-biosensors, because many immune functions are inherently mechanosensitive and are regulated in vivo by vascular perfusion, shear stress, and transport of soluble factors. Compared with static culture of conventional immune-assays, appropriate flow better reproduces key physiological features, such as immune-cell rolling/adhesion on endothelium, chemokine-gradient formation, nutrient/oxygen exchange, and continuous cytokine removal or accumulation, thereby yielding immune responses and biosensor readouts that are more predictive of in vivo behavior. Conversely, non-physiological flow (too low or too high shear, unstable pressure gradients, or excessive confinement) can induce artificial activation or stress responses and may confound interpretation of sensing signals. Therefore, designing and reporting flow regimes that approximate relevant in vivo microenvironments (e.g., microvascular or tissue-specific perfusion levels) is essential for improving biological relevance, reproducibility, and translational value of organ-on-a-chip and microfluidic cell-based immuno-biosensing platforms. Moreover, the immobilization of bioreceptors on transducer surfaces often relies on multi-step chemistries that are difficult to control at scale, with resulting in device-to-device variations in binding capacity and signal response. In addition, harsh regeneration conditions required to dissociate antigen–antibody complexes can damage the biorecognition layer, making many platforms effectively single-use. Long-term operation is further constrained by protein deposition, nonspecific cell adhesion, and particulate contamination that can foul electrode/optical surfaces and clog microchannels, which is particularly problematic for continuous monitoring and single-cell assays. A major limitation for clinical translation is the lack of standardized protocols, reference materials, and calibration/benchmarking procedures. Because studies often report modality-specific and non-uniform endpoints (e.g., LOD for immunoassays versus pairing efficiency or functional activation metrics for live-cell assays) under different cell sources and flow conditions, direct cross-platform performance comparison is difficult and can be misleading. This lack of harmonized reporting and benchmarking hinders reproducibility and complicates the establishment of analytical validity required for regulated diagnostic deployment.

## 7. Future Perspectives

In contrast to traditional immunosensors which depend strictly on antigen–antibody interactions, cell-based microfluidic immuno-biosensors monitor functional and multi-parameter cellular responses that potentially offer a comprehensive and more natural readout under the conditions in vivo. Firstly, improving control of the physical microenvironment, such as implementing physiological flow and shear, stable gradients, and tissue–immune interfaces in organ-on-a-chip formats, will be critical to enhance biological relevance while minimizing mechanically induced artifacts. Secondly, integrating multimodal sensing (e.g., optical with electrical/impedance and electrochemical or mechanical readouts) together with robust computational analysis may improve specificity, mitigate drift, and enable more reliable immune-state metrics, accelerating translation toward clinically actionable applications. For complete patient-specific diagnostics, however, the limited accessibility to primary patient cells as well as their heterogeneous nature are still significant barriers. Immune cells derived from stem cells (e.g., iPSC-derived macrophages, NK cells, dendritic-like cells) present an attractive alternative, providing consistent yet human-relevant sensing systems. Key challenges include phenotype fidelity, batch consistency, and functional drift. A major breakthrough would be standardized quality control combining marker panels with functional calibration to canonical stimuli. When combined with recent advances in optimal gene editing and synthetic biology, next-generation immuno-biosensors can be developed with even more advanced functionality from passive reporters to programmable living sensors. Gene circuits can be engineered to (i) increase specificity by implementing logic-gated recognition of complex inputs (e.g., AND/OR/NOT responses to cytokine combinations or antigen cues), (ii) convert transient immune activation into stable, quantifiable outputs through memory or signal-amplification modules, and (iii) enable built-in self-calibration and safety controls that reduce baseline noise and improve reproducibility. In addition, engineering immune cells with standardized reporters (fluorescent, electrochemical, or secreted signals) can decouple sensing performance from donor variability and facilitate cross-platform benchmarking. Lastly, integrating these platforms into wearable/portable devices could broaden their application to point-of-care and real-time health monitoring. Near-term implementation will likely use disposable microfluidic cartridges with simplified fluid handling (capillary/low-power pumping) and low-power readouts (impedance/electrochemical with minimal optics), coupled to smartphone or handheld analytics. Key hurdles include maintaining cell viability outside incubators, managing calibration/drift in real-world conditions, and embedding standardized controls—advances that would enable frequent immune-state tracking for therapy response, infection risk, and inflammatory flares.

## 8. Conclusions

In conclusion, recent advances in cell-based immuno-biosensors, particularly those enabled by microfluidic technologies, have substantially expanded the scope and relevance of biosensing by harnessing immune cells as active, physiologically faithful recognition elements. Compared with conventional affinity-based immunosensors, these platforms report functional cellular responses to pathogens, tumor cues, or pharmacologic stimuli, thereby offering richer and more clinically representative information. Microfluidic integration has been central to this progress, providing controlled microenvironments, reduced sample and reagent demand, and high-throughput single-cell handling, while supporting diverse transduction strategies and multimodal readouts. At the same time, important barriers remain—most notably maintaining immune-cell viability and stability over extended operation, mitigating chip fouling and fabrication variability, and establishing standardized performance benchmarks necessary for robust clinical deployment. Looking forward, the convergence of organ-on-chip systems, engineered or stem cell-derived immune sensors, single-cell multi-omics, and AI-assisted signal interpretation is expected to further improve sensitivity, specificity, and translational utility. Continued work along these directions should accelerate the maturation of cell-based immuno-biosensors from promising laboratory tools to reliable point-of-care and personalized-medicine technologies.

## Figures and Tables

**Table 1:** Concise comparison of traditional molecular immunosensors vs. cell-based microfluidic immuno-biosensors.

Aspect	Traditional Molecular Immunosensors	Cell-Based Microfluidic Immuno-Biosensors
**Sensing element**	Purified receptors/biomolecules (antibody, aptamer, enzyme)	Living immune cells (primary or engineered)
**Primary output**	Target concentration (single/few biomarkers)	Functional immune response (activation, cytokines, migration, cytotoxicity)
**Molecular level**	High specificity to predefined analytes	Measures integrated pathway responses; can yield multi-parameter response signatures
**Cellular level**	Limited/no cell context; no cell–cell interaction	Captures cell behavior, heterogeneity, and cell–cell interactions (e.g., immune synapse)
**Organ/tissue level**	Typically static, low physiological relevance	Can incorporate flow/gradients/barriers via microfluidics, organ-on-chip, organoids
**Readout modes**	Optical/electrochemical/SPR, often endpoint	Optical + electrical/impedance + electrochemical + mechanical; often real-time
**Strengths**	Standardized, reproducible, clinically mature	Physiologically relevant functional phenotyping; suited for potency and immune-state assessment
**Key limitations**	Limited functional insight; depends on known biomarkers	Biological variability and standardization needs (drift, calibration, validation metrics)

**Table 2:** Categorization of microfluidic immuno-biosensing platforms.

Platform Category	Primary Immuno Targets/Use Cases	Typical Strengths vs. Conventional Immunoassays	Common Readouts	Key Limitations
**Continuous-flow immunoassay chips** [6]	Soluble biomarkers (cytokines, antibodies), antigen/pathogen detection	Low sample/reagent, faster kinetics, automation, portability, multiplexing	Fluorescence/absorbance, electrochemical, impedance	Surface fouling, calibration/standardization, matrix effects, risk of contamination
**Droplet microfluidics** [7]	Single-cell secretion (cytokines), immune activation screening, antibody discovery	True single-cell isolation, high throughput, reduced cross-talk, improved effective concentration	Fluorescence, bead-based capture, imaging/decoding	Device complexity, droplet stability, interfacing to downstream analysis
**Microwell** [8]	Single-cell profiling, ultra-sensitive detection via compartmentalization	Parallelization, improved LOD (digital counting), multiplexing	Fluorescence/chemiluminescence imaging, sometimes electrochemical	Imaging burden, assay workflow complexity, limited long-term culture
**Organ-on-chip** [9]	Inflammation, barrier integrity, migration/extravasation, immunotoxicology	Higher physiological relevance, flow + gradients, dynamic functional readouts	Imaging, TEER/impedance, secretome sampling, integrated sensors	Higher cost/complexity, standardization, longer setup times
**Sorting/enrichment modules** [10]	Rare cell/pathogen enrichment, matrix cleanup	Improved sensitivity/robustness; sample-in/answer-out integration	Acoustic/DEP/inertial/magnetic manipulation, downstream sensing	Device tuning, sample variability, potential cell stress

## Data Availability

No new data were created or analyzed in this study.
